# A collective call to strengthen monitoring and evaluation efforts to support healthy and sustainable food systems: ‘The Accountability Pact’

**DOI:** 10.1017/S1368980022001173

**Published:** 2022-09

**Authors:** Kelly Garton, Vivica Kraak, Jessica Fanzo, Gary Sacks, Stefanie Vandevijvere, Lawrence Haddad, Hannah Brinsden, Amos Laar, Tilakavati Karupaiah, Nasrin Omidvar, William Masters, Inge Kauer, Boyd Swinburn

**Affiliations:** 1 School of Population Health, Faculty of Medical and Health Sciences, The University of Auckland, 22-30 Park Ave, Grafton, Auckland 1023, New Zealand; 2 Department of Human Nutrition, Foods, and Exercise, Virginia Tech, Blacksburg, VA, USA; 3 Berman Institute of Bioethics, Nitze School of Advanced International Studies (SAIS), Bloomberg School of Public Health, Johns Hopkins University, Washington, DC, USA; 4 Global Obesity Centre, Deakin University, Burwood, VIC, Australia; 5 Public Health Nutrition, Epidemiology and Public Health, Sciensano, Brussels, Belgium; 6 The Global Alliance for Improved Nutrition, Geneva, Switzerland; Washington, DC, USA; 7 World Obesity Federation, London, UK; 8 Department of Population, Family and Reproductive Health, School of Public Health, University of Ghana, Accra, Ghana; 9 School of Biosciences, Faculty of Health and Medical Sciences, Taylor’s University, Subang Jaya, Malaysia; 10 Department of Community Nutrition, Faculty of Nutrition Sciences and Food Technology, Shahid Beheshti University of Medical Sciences and Health Services, Tehran, Iran; 11 Friedman School of Nutrition Science and Policy, Tufts University, Boston, MA, USA; 12 Access to Nutrition Initiative, Access to Nutrition Foundation, Utrecht, MJ, The Netherlands

**Keywords:** Accountability, Food systems, Sustainability, Collective action

## Abstract

There is widespread agreement among experts that a fundamental reorientation of global, regional, national and local food systems is needed to achieve the UN Sustainable Development Goals Agenda and address the linked challenges of undernutrition, obesity and climate change described as the Global Syndemic. Recognising the urgency of this imperative, a wide range of global stakeholders – governments, civil society, academia, agri-food industry, business leaders and donors – convened at the September 2021 UN Food Systems Summit to coordinate numerous statements, commitments and declarations for action to transform food systems. As the dust settles, how will they be pieced together, how will governments and food corporations be held to account and by whom? New data, analytical methods and global coalitions have created an opportunity and a need for those working in food systems monitoring to scale up and connect their efforts in order to inform and strengthen accountability actions for food systems. To this end, we present – and encourage stakeholders to join or support – an Accountability Pact to catalyse an evidence-informed transformation of current food systems to promote human and ecological health and wellbeing, social equity and economic prosperity.

Experts and other stakeholders agree that fundamental reorientation of global, regional, national and local food systems is needed to achieve the UN Sustainable Development Goals Agenda^(1–7)^ and address the linked challenges of undernutrition, obesity and climate change described as the Global Syndemic^(8)^.

Recognising the urgency of this imperative, a wide range of global stakeholders – governments, civil society, academia, agri-food industry, business leaders and donors – convened at the September 2021 United Nations Food Systems Summit (UNFSS) to ‘set the stage’ for global food systems transformation to achieve the Sustainable Development Goals by 2030, yielding numerous statements, commitments and declarations for action to transform food systems for people and planet^(9–11)^. Meanwhile, social movements led by indigenous peoples, scholar activists and other civil society groups expressed dissatisfaction with the UNFSS agenda and governance process. The Global People’s Summit on Food Systems emphasised the importance of food sovereignty, agroecology and sustainable production and a human rights approach to ensure adequate, safe, nutritious and culturally appropriate food for all people^(12)^. The UNFSS was followed by the Nutrition for Growth (N4G) Summit in December 2021 with further high-level commitments to translate into action – and accountability – to end malnutrition in all its forms by 2030.

Now that the dust has settled, how will governments, food corporations and other stakeholders/actors be held to account and by whom? Promising actions are the synthesis of the ‘national pathways’ submitted by governments to the UNFSS^(11)^; the Global Nutrition Report team that will assess the N4G Summit’s national and business commitments to ensure that these are specific, measurable, achievable, relevant and time-bound (SMART)^(13)^ and the Food Systems Countdown to 2030 report which tracks and assesses country food system performance^(14)^. The UNFSS Global Coordination Hub leaders expect governments to strengthen their national pathways with milestones and reporting systems to assess global progress by 2023^(11)^.

To support this proposed scale-up of reporting on progress towards food system transformation, we encourage stakeholders to join or support an Accountability Pact coalition to catalyse an evidence-informed transformation of current food systems to promote human and ecological health and wellbeing, social equity and economic prosperity.

## Food systems monitoring and evaluation platforms

Robust systems are needed to monitor and evaluate food systems change to ensure a coordinated and purposeful (rather than piecemeal) approach to transition and transform food systems. Crucial elements to monitor include: food environment dimensions (e.g. availability, affordability, quality, safety and nutrient composition, vendor properties and promotion or marketing); food supply chains (e.g. food production systems and inputs, food storage and loss, distribution and transport, processing and packaging, retail, marketing, purchasing, disposal and waste); their impacts on planetary health (e.g. carbon and water footprints, land use, biodiversity and animal welfare), human nutrition and health, economies and livelihoods and social equity and inclusion^(14)^. Importantly, monitoring and benchmarking of government policies in these domains, as well as commitments and actions of the private sector, is essential. To complement the Global Nutrition Report’s Nutrition Accountability Framework^(13)^, several existing and emerging independent food system monitoring initiatives and platforms can be used within academia and civil society to drive accountability actions^(13–18)^.

## Food systems accountability

Accountability systems establish the mechanisms and processes for gathering information to measure, monitor, analyse and improve the performance of individuals, companies, institutions and governments against voluntary or mandatory standards, and using this information to improve performance^(19)^, and thus are key to achieving desired outcomes^(20)^. They can be internal (i.e. set up and managed within an organisation) or external (e.g. between government and civil society)^(21)^. Accountability entails stakeholders answering to others empowered with authority to assess how well they have achieved specific tasks or goals and to enforce policies, standards or laws to improve desirable actions and outcomes. Such systems are increasingly framed as ‘accountability ecosystems’ where governance actors interact, recognising the non-linear and inherently relational processes permeated by power dynamics and contextual factors^(22)^.

**Fig. 1:**
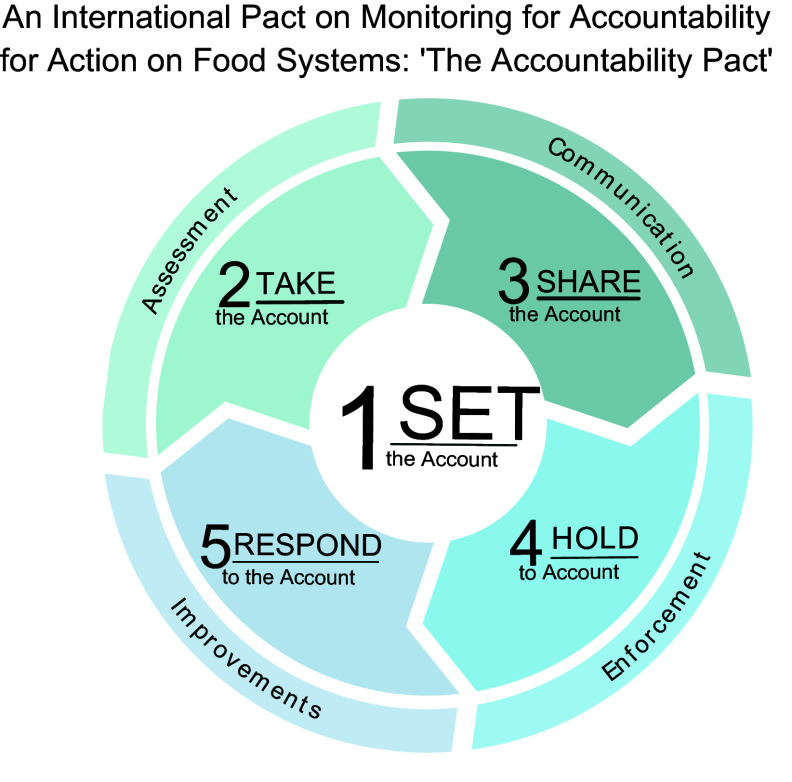
The accountability cycle: an accountability framework for food systems monitoring. Adapted from Kraak *et al.* 2014^(19)^

Robust, independent monitoring and evaluation of food systems can catalyse the necessary transformations using an ‘accountability cycle’ framework to: (1) set the account; (2) take the account; (3) share the account; (4) hold to account and (5) respond to the account (Fig. 1)^(19)^. The key role(s) to be played by the independent research community in each of these stages towards transforming food systems are elaborated below, noting that that there is overlap between these stages and roles. Indeed, the accountability cycle is not a linear process but rather an iterative exercise until desired progress is achieved.

**Setting the account** (stage 1) involves defining the objectives and targets for action.

We need to ‘ask the right questions’ and measure appropriate indicators to *disrupt*, not perpetuate, current food systems. An ambitious agenda should extend across all food system dimensions, taking a systems view recognising that each is interrelated. Setting the account should be participatory and pluralist, inclusive of scientific, local and indigenous knowledge systems^(23)^.

As noted above, high-level commitments must be converted into SMART indicators to enable transparent tracking of progress against the commitments of governments and non-state actors (including the N4G’s six business constituency group members). However, this does little to address the systems issues that did not feature among high-level agendas^(24)^, a notable example being the challenge of curbing the production, distribution, promotion and consumption of ultra-processed foods in the UNFSS^(25)^. Another way to set the account is for researchers and civil society to develop SMART indicators of what *needs to be* done to achieve targets such as the Sustainable Development Goals (i.e. based on recommended targets and actions established by UN agencies and expert consensus bodies). Both approaches are important. The first is needed to support government buy-in to the accountability process, whereas the second may encourage more progressive change. Critically, simply taking country or company commitments at face value and measuring progress towards them is pseudo-accountability that will fail to produce the necessary (and likely uncomfortable) transformations required to achieve healthy and sustainable food systems.

**Taking the account** (stage 2) means measuring the situation and progress towards targets (e.g. monitoring food policies, actions, environments, systems, consumption trends and population and planetary health).

Responsibility for monitoring food systems transformations for accountability should be shared between government and civil society, with participation from businesses to report their commitments and actions. Systematic monitoring of the healthiness and sustainability of national food systems should be a fundamental state responsibility. However, in the absence of systematic government-led monitoring and reporting, independent monitoring and evaluation by civil society and researchers will play a critical role and is especially important with respect to measuring the performance of governments. Independent monitoring, auditing and evaluation of private-sector firms and global coalitions are also essential, particularly given that several aspects of food and beverage industry activity in relation to nutrition, health and environmental sustainability (such as marketing and labelling) are currently poorly regulated globally, with limited mandatory reporting and a lack of consistency in the aspects that are voluntarily reported.

At a global level, there is a need to align existing accountability mechanisms and metrics and address gaps, since each monitoring system has strengths and limitations. The Food Systems Dashboard^(17)^ has organised secondary data from over thirty sources along the food system for all countries and has assembled over 200 indicators, but is somewhat limited by requiring comparable data across countries and does not have, as yet, any subnational dashboards (although these are being developed for eight countries)^(26)^. In contrast, INFORMAS^(15)^ generates detailed food environment data from a smaller set of countries, but has stronger engagement with local experts and actors. The Access to Nutrition Initiative^(16)^ monitors private-sector commitments, practices and disclosure of manufacturers and retailers across seven areas. Tufts University uses diet cost and affordability metrics^(18)^ that target specific food environment dimensions. Critically, *effective collaboration* and a systems approach are needed between groups monitoring different aspects of food environments and levels of food systems, and from different angles, in order for them to be combined in a systematic way.

**Sharing the account** (stage 3) involves communicating the results to decision-makers and other actors by translating and disseminating information from monitoring and evaluation into accessible – and compelling – evidence for action.

A major challenge is ensuring that monitoring and evaluation data are translated for and *received by* policymakers and private-sector actors. First and foremost will be framing and integrating monitoring data with the UN frameworks (e.g. the UN Decade of Action for Nutrition 2016–2025 priorities and the Sustainable Development Goal Agenda goals and targets) and accountability mechanisms that emerged from the UNFSS where possible (though going beyond their scope as appropriate). Here, we argue that there is a growing and important role for ‘scholar activism’ (i.e. academics involved in advocacy for evidence-based policies) coordinated with and led by civil society organisations to hold powerful government and corporate actors accountable for their commitments and actions^(27–29)^. This will necessarily involve civil society activists and researchers strategically communicating evidence using traditional and social media platforms, policy briefings and other digital communications to inform policies and accountability actions. A challenge is that many research teams have unmet funding needs for communication and knowledge exchange and grants often do not support advocacy, although some funders have led the way in supporting strategic communication and knowledge exchange.

**Holding to account** (stage 4) entails providing appropriate incentives and disincentives to drive desired actions from each set of stakeholders.

Though accountability mechanisms are likely to work best when they are constructive (e.g. praising leaders in business or government who have made progress), they are also likely to be most effective when they carry a penalty for actors who undermine healthy and sustainable food systems. Such incentives and disincentives may be legal, financial, political or reputational. While researchers may have limited power in this space, independent scientific research and expertise hold perceived legitimacy. This legitimacy, and information regarding the impact of stakeholder actions and the extent of progress against targets and commitments, can be leveraged by those with power, including the media, to encourage change. For example, investors can have substantial influence over private sector practices and should be encouraged to use a range of strategies (including engagement and investment/divestment) as part of holding to account.

The enabling environment for accountability is as important as the accountability mechanisms themselves. There is a need to navigate and collectively reshape accountability relationships to shift power towards those seeking to demand, enable and enforce public accountability^(22)^. For the research community, this may mean supporting the voices of other actors, including watchdog organisations and investors, to demand, enable and enforce action. Strategies that connect actors, spaces, tools and accountability systems and mechanisms are likely to have a greater chance of achieving and strengthening accountability over time^(22)^.

**Responding to the account** (stage 5) refers to taking actions to improve food systems.

Research is critical to identify and drive the implementation of effective solutions. Researchers can provide valuable evidence and expertise to identify and support the implementation of solutions; for instance, in the design of government or company policies. Data provided to, or generated by, civil society, advocacy coalitions and investors that benchmark commitments and progress achieved can support their efforts by encouraging action.

## The Accountability Pact

It has been argued that the magnitude of the changes required for food systems transformation, and multi-sectoral nature of that transformation, is beyond the scope of existing individual accountability initiatives operating independently^(30)^. We, a global network of independent scientists and food system experts engaged in generating and translating evidence on progress towards healthy and sustainable food systems, have formed ‘The Accountability Pact’: An International Pact on Monitoring for Accountability for Action on Food Systems^(31)^. This is an open network, and we encourage others to join.

To catalyse evidence-informed transformation of current food systems so that they promote ecological health and wellbeing, human health and wellbeing, social equity and economic prosperity, the Accountability Pact endeavours to ensure that all of the necessary elements of the accountability cycle are being met and promotes a coordinated approach across existing platforms. This initiative aspires to: (1) Promote alignment and coherence among accountability mechanisms, to create a comprehensive picture that is greater than the sum of its parts; (2) Evaluate the progress of governments and agri-food and beverage industry towards their SMART commitments from the UNFSS and N4G, and other initiatives for creating healthy, sustainable, equitable and prosperous food systems; (3) Provide robust monitoring data to support the efforts of civil society organisations, investors, funders and other actors advocating for improved food systems, including voices that may have been marginalised in the UNFSS process and (4) Increase accountability literacy within the food systems ‘ecosystem.’

## Conclusions

The global food systems solutions, coalitions, dialogues, commitments and initiatives associated with the UNFSS, N4G Summit and parallel people’s movements will require robust independent monitoring to ensure progress is made towards healthy, fair and sustainable food systems transformations and clear reporting frameworks for governments and food companies. There is an opportunity and need to scale up and connect food systems monitoring to strengthen accountability for action by Member States and the private sector. By bringing various complementary monitoring systems together, learning from each other, expanding the monitoring for accountability efforts and engaging with stakeholders who can integrate accountability data into their advocacy, we can contribute to creating food systems that can better deliver on human health and wellbeing, ecological health and wellbeing, social equity and economic prosperity.
